# STAR LIGHT Study: XBB.1.5 COVID-19 mRNA Vaccines Boost Systemic but Not Mucosal Immunity Against the SARS-CoV-2 JN.1 Variant in Patients with Chronic Liver Disease

**DOI:** 10.3390/vaccines12111241

**Published:** 2024-10-31

**Authors:** Simon Woelfel, Daniel Junker, Irina Bergamin, Pamela Meyer-Herbon, Roman Stillhard, Nicole Graf, Georg Leinenkugel, Joel Dütschler, Marius König, Livia Kammerlander, Rahel Häuptle, Sarah Zwyssig, Claudia Krieger, Samuel Truniger, Seraina Koller, Katline Metzger-Peter, Nicola Frei, STAR SIGN Study Investigators, Werner C. Albrich, Matthias Friedrich, Christine Bernsmeier, Jan Hendrik Niess, Wolfgang Korte, Justus J. Bürgi, Alex Dulovic, Nicole Schneiderhan-Marra, David Semela, Stephan Brand

**Affiliations:** 1 Department of Gastroenterology and Hepatology, Cantonal Hospital St. Gallen, 9007 St. Gallen, Switzerland; 2 Max von Pettenkofer Institute of Hygiene and Medical Microbiology, Faculty of Medicine, Ludwig Maximilian University (LMU), 80336 Munich, Germany; 3 NMI Natural and Medical Sciences Institute at the University of Tübingen, 72770 Reutlingen, Germany; 4 Clinical Trials Unit, Cantonal Hospital St. Gallen, 9007 St. Gallen, Switzerland; 5 University Center for Gastrointestinal and Liver Diseases, St. Clara Hospital and University Hospital of Basel, 4002 Basel, Switzerland; 6 Outpatient Clinic, Ambulatory Services Rorschach, 9400 Rorschach, Switzerland; 7 Division of Infectious Diseases, Infection Prevention and Travel Medicine, Cantonal Hospital St. Gallen, 9007 St. Gallen, Switzerland; 8 Translational Gastroenterology and Liver Unit, Nuffield Department of Medicine, University of Oxford, Oxford OX3 9DU, UK; 9 Gastroenterology Group, Department of Biomedicine, University of Basel, 4031 Basel, Switzerland; 10 Center for Laboratory Medicine, 9001 St. Gallen, Switzerland

**Keywords:** COVID-19, SARS-CoV-2, mRNA vaccines, XBB.1.5 vaccines, chronic liver disease, liver transplantation, liver cirrhosis, autoimmune hepatitis, inflammatory bowel disease, IBD, SARS-CoV-2 neutralization, mucosal immunogenicity, XBB.1.5, EG.5.1, BA.2.86, JN.1

## Abstract

Background: Patients with chronic liver disease (CLD) have impaired vaccine immunogenicity and an excess risk of severe COVID-19. While variant-adapted COVID-19 mRNA vaccines are recommended for vulnerable individuals, their efficacy in patients with CLD has not been studied. Methods: We present the first evaluation of XBB.1.5 COVID-19 vaccine immunogenicity against the SARS-CoV-2 JN.1 variant in patients with CLD. Serum anti-receptor binding domain (RBD) IgG, neutralization, and saliva anti-RBD IgG and IgA against wild-type SARS-CoV-2 (WT) and the XBB.1.5, EG.5.1, BA.2.86, and JN.1 variants were quantified before and 2–4 weeks following a fourth dose of XBB.1.5 mRNA vaccines. Results: Vaccination boosted anti-RBD IgG and neutralization against all tested variants including JN.1 (each *p* < 0.001). Following immunization, neutralization was lower against JN.1 compared to WT, XBB.1.5, and EG.5.1 (*p* < 0.001, *p* < 0.001, and *p* < 0.01, respectively). Vaccination reduced neutralization failure rates against BA.2.86 and JN.1 (each *p* < 0.05). The evasion of vaccine-induced antibodies by the tested variants was low, indicated by the positive correlation between anti-RBD IgG and neutralization. At mucosal sites, vaccination boosted anti-RBD IgG (each *p* < 0.01) but failed to induce infection-blocking IgA (each *p* > 0.05). Conclusion: XBB.1.5 vaccines protect CLD patients against recent SARS-CoV-2 variants, but developing vaccines with optimized mucosal immunogenicity is required to prevent SARS-CoV-2 transmission and recurrent seasonal COVID-19 outbreaks.

## 1. Introduction

The widespread use of mRNA vaccines saved millions of lives during the COVID-19 pandemic, and vaccines continue to protect from severe disease and infection-associated long-term ailments [1,2,3]. However, vaccine-induced immune responses wane over time and fail to prevent virus transmission entirely [4,5]. In the summer of 2024, the emergence of highly immune-evasive SARS-CoV-2 variants—such as the JN.1 descendants KP.2 and KP.3—sparked one of the highest COVID-19 summer waves since the beginning of the pandemic, leading to millions of infections worldwide [6]. In addition to the risk of severe outcomes such as hospitalization and death, every infection increases the risk of long COVID, which is associated with low levels of protective anti-SARS-CoV-2 antibodies [7,8]. Therefore, it is essential to maintain high levels of immunoprotection during ongoing infection surges. Particular attention is mandated for at-risk individuals who have reduced immunoprotection due to pre-existing health conditions and are currently disproportionately impacted by COVID-19 [9].

This includes patients with chronic liver disease (CLD), a widespread pathology spectrum with diverse etiologies, which often results in liver cirrhosis, fibrosis, cancer, or transplantation [10]. Due to cirrhosis-mediated immune dysfunction, CLD patients frequently face an increased risk for infectious diseases, including SARS-CoV-2 infections [11,12]. Recent studies demonstrated that CLD patients have an increased risk of severe COVID-19, as indicated by higher rates of hospitalization and ventilation in CLD compared to non-CLD COVID-19 patients [12,13]. Moreover, several CLDs, including alcohol-related liver disease, liver cirrhosis, and hepatocellular carcinoma, lead to increased mortality in COVID-19 patients [13,14,15,16]. Susceptibility to SARS-CoV-2 infection and illness is exacerbated by impaired humoral and T-cell-mediated immunogenicity following SARS-CoV-2 vaccination in patients with immunosuppression, liver transplantation, or cirrhosis [17,18,19,20]. Collectively, these data suggest an elevated risk of CLD patients during the current surge in COVID-19 infections.

Therefore, updated COVID-19 vaccines encoding the spike protein of the omicron XBB.1.5 lineage were recommended as booster doses after primary vaccination for patients with CLD, including those with liver cirrhosis, liver transplantation, hepatic cancer, and immunosuppression. Given that recent SARS-CoV-2 variants are direct descendants of XBB.1.5, one may assume that XBB.1.5 vaccines protect against currently circulating SARS-CoV-2 lineages, including the JN.1 subvariant KP.3.1.1, which shows high potential of immune evasion paired with high infectivity [21,22]. However, to date, data on the immunogenicity of XBB.1.5 vaccines such as BNT162b2 XBB.1.5 (BioNTech/Pfizer Corminaty Omicron XBB.1.5) or mRNA-1273.815 (Moderna Spikevax XBB.1.5) in CLD patients is lacking. Therefore, whether such vaccines can overcome poor immunoprotection in CLD patients and foster sufficient neutralization against highly immune-evasive omicron lineages that dominate current infection waves remains obscure.

This collaborative study between three Swiss tertiary hepatology centers, the University of Tuebingen, and the University of Oxford, is the first study to evaluate the efficacy of XBB.1.5 vaccines in patients with CLD. We provide the first comprehensive analysis of the following:

(i) Systemic levels of variant-specific anti-receptor binding domain (RBD) IgG and serum-mediated inhibition of ACE2 binding by wild-type SARS-CoV-2 and the XBB.1.5, EG.5.1, BA.2.86, and JN.1 lineages before and after receiving an XBB.1.5 mRNA vaccine as a fourth vaccine dose;
(ii) Mucosal levels of anti-RBD IgG and IgA before and after receiving an XBB.1.5 mRNA vaccine as a fourth vaccine dose.

## 2. Materials and Methods

### 2.1. Study Design, Participant Recruitment, and Study Procedures

The STAR LIGHT (**S**ys**T**emic and mucosal S**AR**S-CoV-2 vaccine responses in patients with **LI**ver transplantation, liver cirrhosis, and mali**G**nant **H**epatic **T**umors) study is a prospective longitudinal multicenter cohort study that aims to evaluate the impact of chronic liver disease on immune responses to COVID-19 vaccines. Study approval was obtained by the responsible ethics committee together with the STAR SIGN study [23,24].

Sites of participant recruitment were the outpatient clinics of the Cantonal Hospital St. Gallen, Gastroenterology and Hepatology Clinic Rorschach, and the University Digestive Health Care Center, Clarunis, Basel. The study inclusion criteria were age of 18 years or older, diagnosis of CLD (liver transplantation, liver cirrhosis, or non-cirrhotic chronic liver disease), and triple vaccination with the original COVID-19 mRNA vaccines. The study exclusion criteria were SARS-CoV-2 infection within six months before study inclusion or during participation and pregnancy at study inclusion or during participation.

Study procedures were performed during two study visits at the respective outpatient clinic. At visit 1, study procedures included recording baseline characteristics via a study questionnaire, collecting serum and saliva samples, and vaccination with XBB.1.5 mRNA vaccines—either BNT162b2 XBB.1.5 (BioNTech/Pfizer Corminaty Omicron XBB.1.5) or mRNA-1273.815 (Moderna Spikevax XBB.1.5). Visit 2 was two to four weeks after visit 1 and included recording vaccine-mediated adverse events via a study questionnaire and collecting serum and saliva samples.

### 2.2. Immunochemical Assays

Serum and saliva samples were used to evaluate systemic and mucosal immune responses to vaccination. The MULTICOV-AB immunoassay was used to quantify serum and saliva anti-RBD IgG and IgA levels against SARS-CoV-2 variants. This is a bead-based multiplex assay allowing for the paralleled quantification of antibodies targeting different antigens. Antibody levels are presented as mean fluorescence intensity (MFI) values. This unit strongly correlates with binding antibody units per mL—the predominant unit for reporting antibody levels—in a non-linear, near-logarithmic way. The detailed methodology of this technique has been described previously [25].

Neutralization of SARS-CoV-2 variants by serum neutralizing antibodies was assessed using the RBDCoV-ACE2 immunoassay, which is a clinically validated bead-based multiplex assay that measures the extent to which serum-contained neutralizing antibodies can block the interaction between the SARS-CoV-2 RBD and the human ACE2 receptor. Methodological details and a description of the underlying principle have been published previously [26,27]. Samples with neutralization below 20% are non-neutralizing, which has been validated before using live virus neutralization assays [26].

### 2.3. Study Outcomes

The primary outcomes were anti-RBD IgG serum levels and neutralization against wild-type SARS-CoV-2 and the XBB.1.5, EG.5.1, BA.2.86, and JN.1 variants before and two to four weeks after vaccination with XBB.1.5 COVID-19 mRNA vaccines.

The secondary outcomes were as follows:

- Saliva levels of anti-RBD IgA and IgG against wild-type SARS-CoV-2 and the XBB.1.5, EG.5.1, BA.2.86, and JN.1 variants before and two to four weeks after vaccination;
- Adverse events reported within seven days following vaccination.

### 2.4. Statistical Analysis

For categorical and continuous variables in Table 1, absolute and relative frequencies and mean and standard deviations (SD) are presented, respectively. The results of dependent samples, including the comparisons of virus neutralization, anti-RBD IgG, and IgA levels before and after vaccination, were analyzed using the exact Wilcoxon signed-rank test. Multiple testing of dependent samples, including comparing immune responses against different omicron variants was performed using Friedman tests. The results of independent variables, including the comparisons of virus neutralization, anti-RBD IgG, and IgA levels between the different study groups, were analyzed using the exact Mann–Whitney test with Dunn’s correction. Proportions of non-neutralizing individuals and correlations between variables were analyzed using Fisher’s exact test and Spearman’s rank correlation, respectively. All statistical tests were performed using GraphPad Prism version 9.3.1 or R version 4.2.2.

## 3. Results

### 3.1. Study Population and Adverse Events

Between November 2023 and February 2024, more than 200 patients with CLD from three Swiss tertiary IBD centers were screened for study eligibility. Of these, twenty patients fulfilled the stringent study inclusion criteria and consented to participation in the STAR LIGHT study. Participants had a mean age of 62.1 years (SD 10.7 years), and 35% were female (Table 1). Seven of twenty participants (35.0%) were liver transplant patients, 9 (45.0%) had liver cirrhosis, and four (20.0%) had non-cirrhotic chronic liver disease. Eighteen participants (90%) received BNT162b2 XBB.1.5 and two (10%) received mRNA-1273.815 as a fourth vaccine dose. Local and systemic adverse events in response to vaccination were observed in 35% and 25% of participants, respectively (Table S1).

### 3.2. Serum Antibodies and Neutralizing Immunity Against Omicron Subvariants Are Induced by XBB.1.5 COVID-19 mRNA in Patients with Chronic Liver Disease

**Figure 1:**
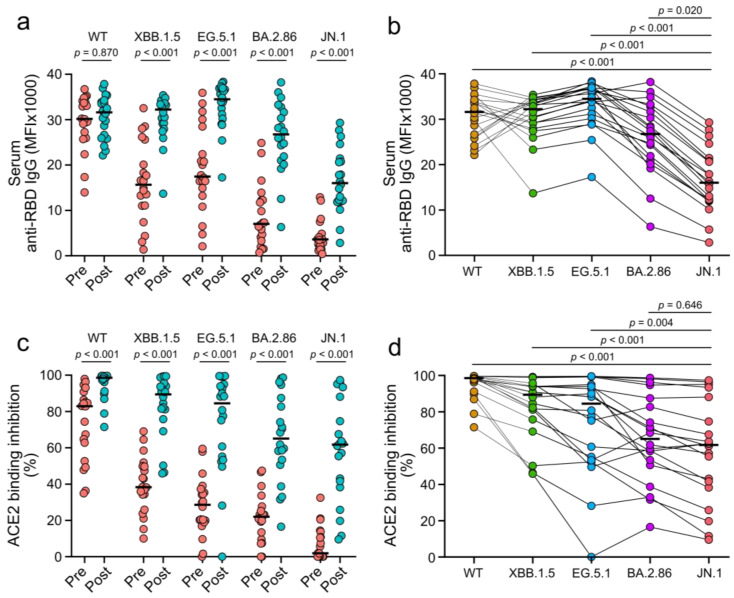
Systemic and neutralizing immune responses against wild-type SARS-CoV-2 (WT) and omicron subvariants induced by XBB.1.5 COVID-19 vaccines. (**a**) Serum levels of anti-RBD IgG before (pre) and two to four weeks after (post) vaccination. (**b**) Stratification of anti-RBD IgG levels two to four weeks after vaccination by virus variant. (**c**) Serum-mediated ACE2 binding inhibition before (pre) and two to four weeks after (post) vaccination. (**d**) Stratification of ACE2 binding inhibition two to four weeks after vaccination by virus variant. Crossbars indicate medians and statistical analyses are based on exact Wilcoxon signed-rank tests (**a**,**c**) or Friedman tests (**b**,**d**).

The levels of serum anti-receptor binding domain (RBD) IgG against omicron variants were higher two to four weeks after vaccination compared to pre-vaccination (each *p* < 0.001; Figure 1a). This was not the case for anti-RBD IgG-targeting wild-type SARS-CoV-2 (*p* = 0.870). The highest increase in antibody levels was observed for anti-RBD IgG-targeting JN.1 (4.4-fold), followed by antibodies targeting BA.2.86 (3.8-fold), XBB.1.5 (2.1-fold), EG.5.1 (2.0-fold), and wild-type (1.0-fold) SARS-CoV-2. Following vaccination, levels of anti-RBD IgG targeting the JN.1 variant were lower than levels of antibodies targeting any of the other tested SARS-CoV-2 lineages (each *p* ≤ 0.020; Figure 1b). The neutralization capacity of patient sera against omicron subvariants was evaluated to assess the functional impact of vaccine-induced antibodies. This was performed using serum-mediated inhibition of RBD-ACE2 binding as a surrogate, which reliably matches the results of live virus neutralization assays [26]. ACE2 binding inhibition was higher two to four weeks after vaccination compared to pre-vaccination (each *p* < 0.001; Figure 1c). Following vaccination, serum-mediated inhibition of ACE2 binding by the JN.1 variant was lower than inhibition of ACE2 binding by the EG.5.1 and XBB.1.5 variants and wild-type SARS-CoV-2 (each *p* ≤ 0.004; Figure 1d). No difference in anti-RBD IgG levels and ACE2 binding inhibition for any tested lineage was observed between patients with liver cirrhosis and those with liver transplantation (each *p* ≥ 0.091; Figure S1). These results suggest that XBB.1.5 vaccines induce RBD-specific and neutralizing antibodies that target different omicron subvariants and can be partially evaded by the JN.1 variant.

### 3.3. Omicron Subvariant Neutralization Following XBB.1.5 COVID-19 Vaccines Is Partially Lacking in Patients with Chronic Liver Disease and Correlates with anti-RBD IgG Levels

**Figure 2:**
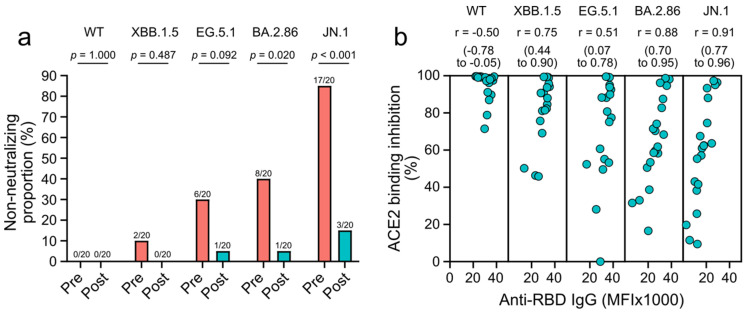
Neutralization failure against wild-type SARS-CoV-2 (WT) and omicron subvariants, and correlation of neutralization and antibody responses following XBB.1.5 vaccines. (**a**) Proportions of individuals lacking neutralization against the indicated SARS-CoV-2 variants before (pre) and two to four weeks after (post) vaccination. (**b**) Correlations of antibody and neutralizing responses targeting wild-type SARS-CoV-2 (WT) and omicron subvariants, two to four weeks after vaccination. Statistical analyses are based on Fisher’s exact tests (**a**) and Spearman’s rank correlations with 95% confident intervals (**b**).

To assess if XBB.1.5 mRNA vaccines sufficiently protect patients with CLD against immune-evasive SARS-CoV-2 variants, we calculated the proportions of individuals who were lacking neutralization, as indicated by ACE2 binding inhibition of less than 20% (see methods section) [26]. Proportions of neutralization failure against the BA.2.86 and JN.1 variants were lower after vaccination with XBB.1.5 vaccines compared to pre-vaccination (each *p* ≤ 0.020; Figure 2a). However, even after vaccination, 5%, and 15% of participants lacked neutralization against the BA.2.86 and JN.1 variants, respectively. Similarly, 5% lacked neutralization against the EG.5.1 variant. Proportions of neutralization failure against wild-type SARS-CoV-2 and the XBB.1.5 variant were low even before vaccination (0% and 10%) and were 0% after immunization (Figure 2a). ACE2 binding inhibition following vaccination with XBB.1.5 vaccines positively correlated with levels of anti-RBD IgG for all tested omicron subvariants (each r ≥ 0.51) but not for wild-type SARS-CoV-2 (r = −0.50; Figure 2b). These results suggest that vaccine-elicited antibodies neutralize the tested omicron subvariants and that several patients with CLD lacked neutralization against the JN.1 variant.

### 3.4. XBB.1.5 COVID-19 Vaccines Fail to Induce Mucosal IgA Responses in Patients with Chronic Liver Disease

**Figure 3:**
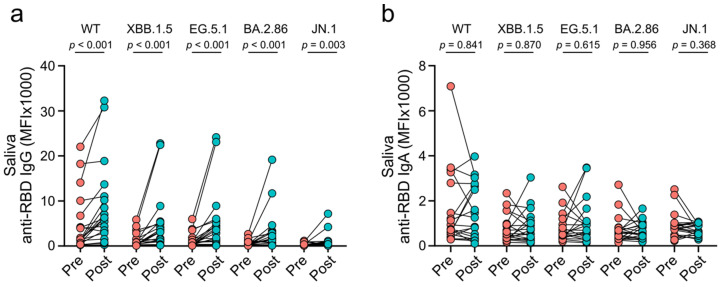
Mucosal antibody responses against wild-type SARS-CoV-2 (WT) and omicron subvariants induced by XBB.1.5 mRNA vaccines. (**a**) Saliva levels of anti-RBD IgG before (pre) and two to four weeks after (post) vaccination. (**b**) Saliva levels of anti-RBD IgA before (pre) and two to four weeks after (post) vaccination. Statistical analyses are based on exact Wilcoxon signed-rank tests.

Mucosal antibodies target SARS-CoV-2 at its entry site into the human body, blocking infection and onward transmission [28,29,30]. To evaluate the effect of XBB.1.5 vaccines on mucosal immune responses, we quantified saliva levels of anti-RBD IgG and IgA before and after vaccination. Saliva levels of anti-RBD IgG against all tested SARS-CoV-2 lineages were higher after immunization than pre-vaccination (each *p* ≤ 0.003; Figure 3a). However, no difference was observed for anti-RBD IgA against any of the tested SARS-CoV-2 lin-eages when comparing saliva levels post- and pre-vaccination (each *p* ≥ 0.368; Figure 3b). Following vaccination, levels of anti-RBD IgA were lower than the respective IgG levels in saliva and serum, except for saliva antibodies against the BA.2.86 and JN.1 variants (each *p* ≤ 0.002; saliva BA.2.86: *p* = 0.091; saliva JN.1: *p* > 0.331; Figure S2). Serum and saliva anti-RBD IgG levels targeting the same omicron variants showed a moderate to strong positive correlation, while no correlation was observed for the respective IgA levels (IgG: each r ≥ 0.58; IgA: each r ≤ 0.33; Figure S3). These data indicate that XBB.1.5 vaccines induce mucosal IgG responses against SARS-CoV-2 but fail to induce mucosal IgA responses in patients with CLD.

### 3.5. XBB.1.5 COVID-19 Vaccines Induce Similar Immune Responses in Patients with Chronic Liver Disease and Healthy Controls

We recently evaluated XBB.1.5 COVID-19 vaccine-elicited immune responses in patients with inflammatory bowel disease (IBD) on biologic therapy targeting TNF (anti-TNF) or other cellular targets (non-anti-TNF) [31,32]. Numerous studies have shown that non-anti-TNF-treated patients with IBD have equal immune responses following COVID-19 vaccination as healthy individuals [23,24,31,33,34]. Therefore, our data from non-anti-TNF-treated patients with IBD can be used as a proxy for immune responses in healthy individuals. Following immunization with XBB.1.5 vaccines, patients with CLD had comparable anti-RBD IgG serum levels and ACE2 binding inhibition to non-anti-TNF-treated patients with IBD (*p* > 0.999 for all tested SARS-CoV-2 lineages; Figure S4). In contrast, anti-TNF-treated patients with IBD, known to have impaired vaccine immunogenicity [23,24,31,33,34], had reduced inhibition of ACE2 binding by all tested SARS-CoV-2 lineages compared to patients with CLD (each *p* ≤ 0.048; Figure S4). These results suggest that patients with CLD may have similar immunogenicity towards XBB.1.5 COVID-19 vaccines as healthy controls.

## 4. Discussion

The emergence of highly immune-evasive SARS-CoV-2 variants in 2024 sparked one of the largest COVID-19 summer waves since the beginning of the pandemic. Variant-adapted COVID-19 vaccines are available, but undervaccination remains common [35]. In our study, XBB.1.5 COVID-19 vaccines seemingly elicited robust systemic humoral and neutralizing immunity in patients with CLD, suggesting that adapted vaccines might offer improved protection against the highly immune-evasive JN.1 variant. However, the vaccines fail to induce mucosal IgA responses. Our results align with research in healthy individuals showing that XBB.1.5 vaccines boost JN.1-directed neutralization [36]. Neutralizing antibodies are a robust correlate of protection against COVID-19 [37]. However, the rapid waning of immune responses indicates that this protection only lasts for a limited time before booster vaccination becomes necessary [4].

While infections with recent SARS-CoV-2 variants are mostly mild, the risk of long COVID increases with every additional infection, even in fully vaccinated individuals [7]. Importantly, COVID-19 vaccines were shown to protect against thromboembolic complications, cardiovascular disease, and cognitive impairment associated with post-acute sequelae of COVID-19 [3,38,39]. Therefore, it remains essential to keep up with vaccinations to prevent long-term ailments associated with SARS-CoV-2 infection.

The COVID-19 vaccine tested in this study is tailored to the spike protein of the XBB.1.5 variant. EG.5.1, BA.2.86, and JN.1 are all descendants from XBB.1.5 with JN.1 showing the most mutations compared to XBB.1.5. Our findings on systemic vaccine responses suggest that the JN.1 variant can partially evade neutralizing antibodies elicited by XBB.1.5 vaccines. This aligns with previous findings and likely explains why several study participants lacked JN.1 neutralization even after vaccination. Our findings also demonstrate that XBB.1.5 vaccines are less effective against the JN.1 variant than previous omicron lineages, aligning with studies in healthy individuals [40,41,42]. Frequent booster vaccinations with variant-adapted vaccines will be essential to restore immune protection against immune-evasive SARS-CoV-2 variants [43]. Recently, the FDA approved new COVID-19 vaccines tailored to the JN.1 or KP.2 variants. It will be interesting to see if these updated vaccines elicit more robust neutralization against JN.1 and the currently circulating KP.3.1.1 variant [22,44]. Future studies should also assess SARS-CoV-2 infection rates in vaccinated individuals to evaluate the real-world efficacy of variant-adapted COVID-19 vaccines.

By comparing vaccine responses in CLD and non-anti-TNF-treated patients with IBD, we show that immune responses in the evaluated study population are comparable to those expected in healthy individuals. The inclusion of healthy individuals in our study was hindered by COVID-19 vaccine recommendations in Switzerland which only recommend vaccination to at-risk groups but not healthy individuals. Future studies are required to compare immune responses elicited by variant-adapted vaccines in patients with CLD and healthy individuals. Previous studies showed that both liver transplantation and liver cirrhosis are associated with reduced immunogenicity after original mRNA vaccines [18,19]. This discrepancy between this and previous studies might be explained by the substantially lower severity of liver cirrhosis, as demonstrated by the MELD score of our study participants compared to previous studies. Importantly, our results suggest that in addition to non-CLD-related factors, the severity of liver disease may determine a patient’s predisposition for impaired vaccine immunogenicity.

The current COVID-19 situation resembles an ongoing arms race between humans and the virus. The tailoring of vaccines to newly emerging SARS-CoV-2 variants allows boosting of variant-specific immunity that wanes rapidly and is ultimately evaded through evolutionary adaptation of the viral spike protein. Our finding that currently used mRNA vaccines fail to induce mucosal IgA responses—potentially rendering them ineffective in preventing SARS-CoV-2 transmission—highlights the need to invest in developing novel vaccination strategies. This is supported by previous research showing that current vaccines, while protecting against severe COVID-19, fail to prevent SARS-CoV-2 infections with omicron lineages [45]. Therefore, boosting transmission-blocking immune responses, including mucosal IgA, remains one of the key challenges at this point in time and can potentially end the seasonal recurrence of COVID-19 outbreaks [28,29,46,47,48]. Some promising mucosal vaccine candidates are currently being evaluated, and already demonstrate robust boosting of mucosal IgA and T-cell responses to block SARS-CoV-2 transmission and prevent infection upon exposure [48,49,50].

We acknowledge the small sample size as a limitation of our study. Despite enormous efforts undertaken during study recruitment, the screening of several hundred patients resulted in the recruitment of 20 participants. Since unwillingness to get vaccinated was among the most common reasons for unsuccessful recruitment, the vaccination rate of at-risk patients with CLD is likely far from sufficient. However, our conclusions seem reasonable given that vaccination boosted systemic immunity in 100% of patients and failed to boost mucosal IgA in most. More extensive studies are required to confirm these results and assess the impact of chronic liver disease etiology in more detail.

## 5. Conclusions

This is the first study to evaluate systemic and mucosal immunogenicity of variant-adapted COVID-19 mRNA vaccines in patients with CLD. We report that XBB.1.5 COVID-19 mRNA vaccines induce robust systemic humoral and neutralizing immunity against several omicron lineages including the JN.1 variant. However, they fail to induce SARS-CoV-2-reactive IgA at mucosal sites, which is required for blocking SARS-CoV-2 transmission. Taken together, this study supports the use of variant-adapted COVID-19 mRNA vaccines in patients with CLD and highlights the need for mucosal vaccines to prevent SARS-CoV-2 infection surges.

## Figures and Tables

**Table 1:** Study population baseline characteristics.

	Study Population (*n* = 20)
Age, years (SD)	62.1 (10.7)
Sex (%) Female Male Other	7 (35.0) 13 (65.0) 0 (0.0)
BMI, kg/m^2^ (SD)	26.2 (4.4)
Ethnicity (%) European Asian African Others	20 (100.0) 0 (0.0) 0 (0.0) 0 (0.0)
Smoking status (%) Never Former Current	7 (35.0) 7 (35.0) 6 (30.0)
Diagnosis (%) Liver transplantation Liver cirrhosis Non-cirrhotic chronic liver disease (hepatocellular carcinoma or autoimmune hepatitis)	7 (35.0) 9 (45.0) 4 (20.0)
Duration of disease, days (SD)	1825.2 (1539.9)
MELD score (SD); n = 8	12 (4)
Therapy within the last six months (%) Azathioprine 6-Mercaptopurine Tacrolimus Methotrexate Checkpoint and VEGF inhibitors	1 (5.0) 1 (5.0) 7 (35.0) 0 (0.0) 3 (15.0)
Underlying disease (%) Cancer Heart disease Hypertension Pulmonary disease Kidney disease Diabetes mellitus Arthritis Intestinal disease Hyperlipidemia	5 (25.0) 2 (10.0) 11 (55.0) 1 (5.0) 1 (5.0) 5 (25.0) 1 (5.0) 2 (10.0) 0 (0.0)
SARS-CoV-2 infection since third vaccination (%)	4 (20.0)
Number of SARS-CoV-2 infections ever (%) 0 1 2 3	11 (55.0) 8 (55.0) 0 (0.0) 1 (5.0)
Type of fourth dose vaccine BNT162b2 XBB.1.5 mRNA-1273.815	18 (90.0) 2 (10.0)
Vaccination schedule doses 1–4 (%) Homologous Heterologous	11 (55.0) 9 (45.0)

## Data Availability

Data may be provided upon reasonable request to the corresponding author. In accordance with data sharing regulations outlined in the study protocol, only anonymized raw data can be made available, and any additional analyses of study data cannot deviate from the approved study protocol. Any data transfer requires written consent to a transfer agreement.

## Supplementary Materials

The following supporting information can be downloaded at https://www.mdpi.com/article/10.3390/vaccines12111241/s1, Figure S1: Comparison of immune responses in patients with liver cirrhosis and liver transplantation, induced by XBB.1.5 COVID-19 vaccines; Figure S2: Comparison of IgA and IgG responses at systemic and mucosal sites, induced by XBB.1.5 COVID-19 vaccines; Figure S3: Correlation of systemic and mucosal humoral immune responses, induced by XBB.1.5 COVID-19 vaccines; Figure S4: Comparison of immune responses in patients with chronic liver disease and inflammatory bowel disease, induced by XBB.1.5 COVID-19 vaccines; Table S1: Adverse events triggered by XBB.1.5 COVID-19 mRNA vaccines; Table S2: STAR SIGN study investigators.
