# Supplementary material for: STAR LIGHT Study: XBB.1.5 COVID-19 mRNA Vaccines Boost Systemic but Not Mucosal Immunity Against the SARS-CoV-2 JN.1 Variant in Patients with Chronic Liver Disease
Source: Vaccines (Basel). 2024 Oct 31;12(11):1241. doi: 10.3390/vaccines12111241 (PMC11598625; doi:10.3390/vaccines12111241)
Supplement: Supplementary file 1 [file vaccines-12-01241-s001.zip › vaccines-3253298-supplementary.pdf]

vaccines

MDPI

Supplementary Materials

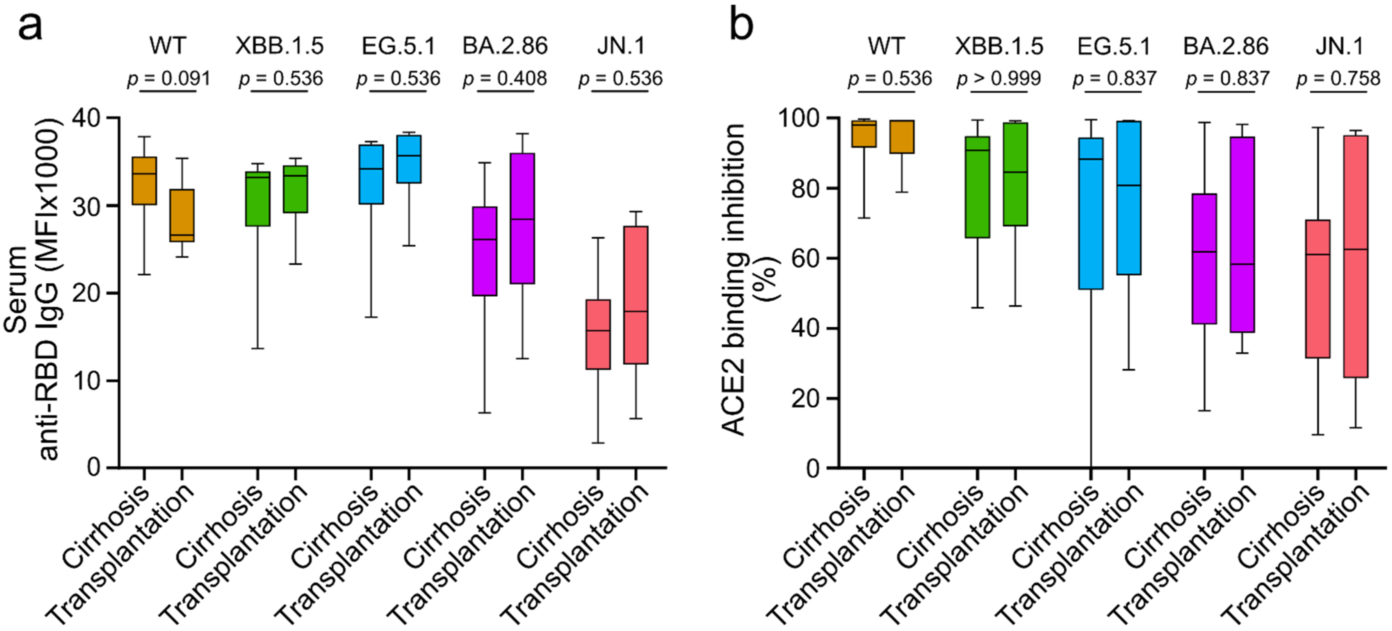

Figure S1: Comparison of immune responses in patients with liver cirrhosis and liver transplantation, induced by XBB.1.5-adapted COVID-19 vaccines. (**a**) Serum levels of anti-RBD IgG against wild-type SARS-CoV-2 (WT) and omicron subvariants two to four weeks after vaccination. (**b**) Serum-mediated inhibition of ACE2 binding by wild-type SARS-CoV-2 (WT) and omicron subvariants two to four weeks after vaccination. Median and interquartile range are indicated by boxes, whiskers indicate minimum and maximum values. Statistical analyses are based on exact Mann-Whitney tests.

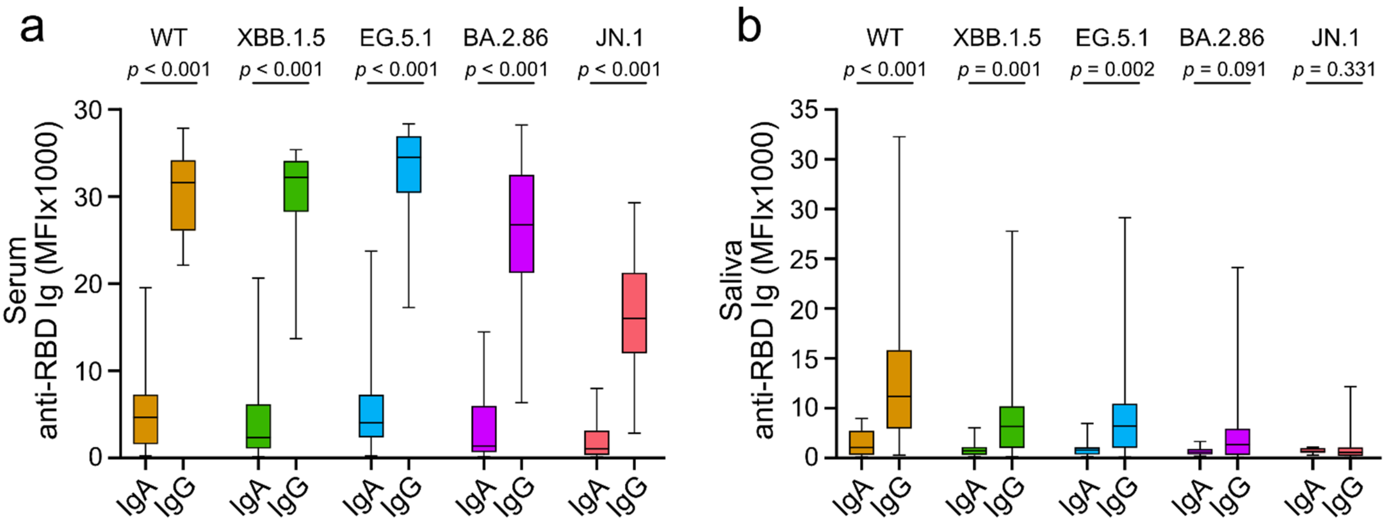

Figure S2: Comparison of IgA and IgG responses at systemic and mucosal sites, induced by XBB.1.5-adapted COVID-19 vaccines. (**a**) Serum levels of anti-RBD IgA and IgG against wild-type SARS-CoV-2 (WT) and omicron subvariants two to four weeks after vaccination. (**b**) Saliva levels of anti-RBD IgA and IgG against wild-type SARS-CoV-2 (WT) and omicron subvariants two to four weeks after vaccination. Median and interquartile range are indicated by boxes, whiskers indicate minimum and maximum values. Statistical analyses are based on exact Mann-Whitney tests.

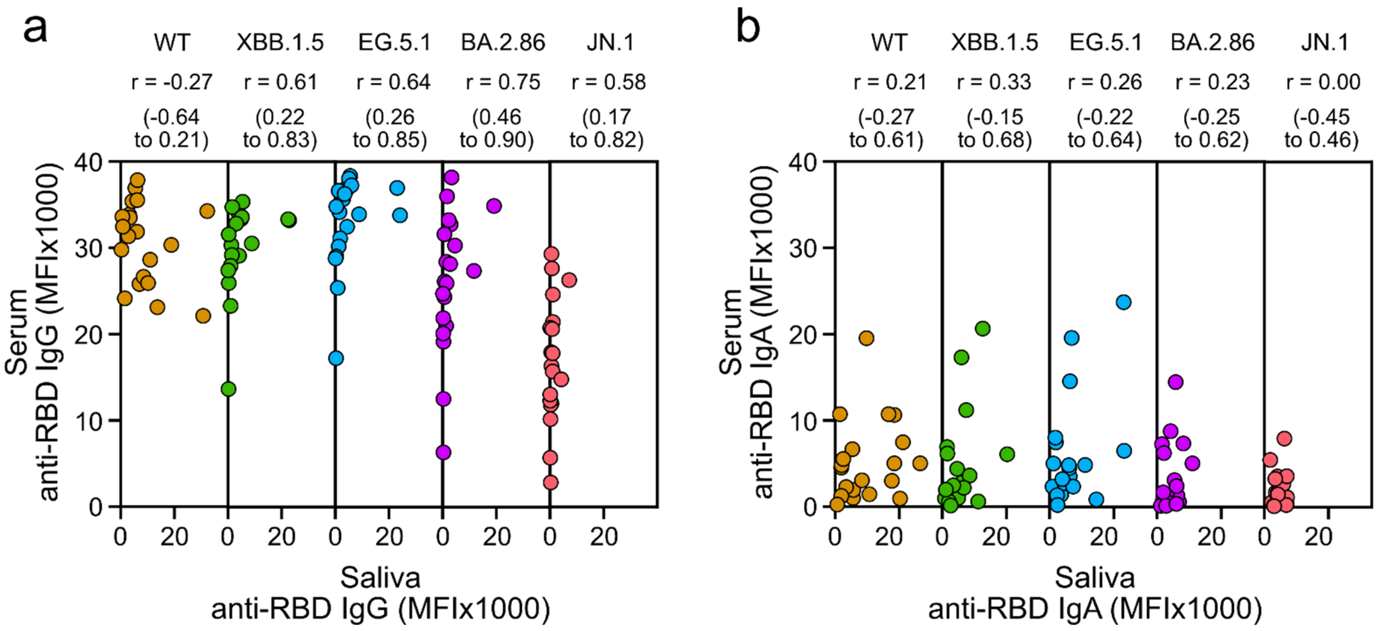

Figure S3: Correlation of systemic and mucosal humoral immune responses, induced by XBB.1.5-adapted COVID-19 vaccines. (a) correlation between serum and saliva levels of IgG against wild-type (WT) and omicron subvariants two to four weeks after vaccination. (b) Correlation between serum and saliva levels of IgA against wild-type (WT) and omicron subvariants two to four weeks after vaccination. Statistical analyses are based on Spearman's rank correlations with 95% confident intervals.

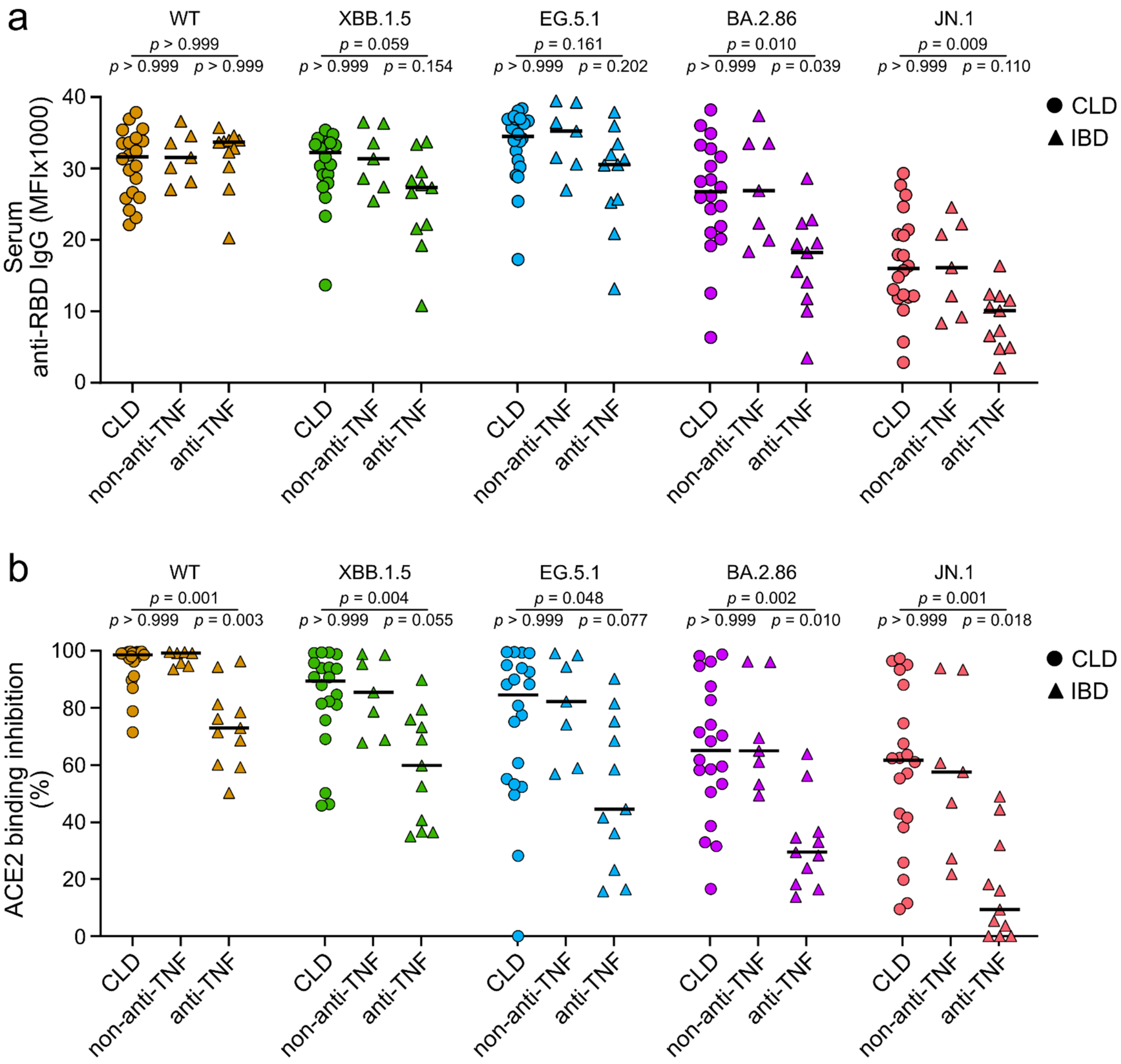

Figure S4: Comparison of immune responses in patients with chronic liver disease and inflammatory bowel disease, induced by XBB.1.5-adapted COVID-19 vaccines. (**a**) Serum levels of anti-RBD IgG against wild-type SARS-CoV-2 (WT) and omicron subvariants two to four weeks after vaccination. (**b**) Serum-mediated inhibition of ACE2 binding by wild-type SARS-CoV-2 (WT) and omicron subvariants two to four weeks after vaccination. Medians are indicated by crossbars and statistical analyses are based on Kruskal Wallis tests with Dunn's correction. IBD cohort data have been published elsewhere (see references 31 and 32 of the main text).

Table S1. Adverse events triggered by XBB.1.5-adapted COVID-19 mRNA vaccines.

	**Study population (n = 20)**
Local adverse events (%)	
Any	7 (35.0)
Pain	6 (30.0)
Erythema	0 (0.0)
Local swelling	1 (5.0)
Axillary swelling	0 (0.0)
Systemic adverse events (%)	
Any	5 (25.0)
Fever	1 (5.0)
Headache	0 (0.0)
Fatigue	3 (15.0)
Myalgia	2 (10.0)
Arthralgia	2 (10.0)
Nausea	0 (0.0)
Chills	1 (5.0)

Table S2. STAR SIGN study investigators.

**First name**	**Last name**
Benjamin	Misselwitz[1,2]
Vasileios	Oikonomou[1]
Jacqueline	Wyss[1]
Niklas	Krupka[1]
Reto	Bertolini[3]
Jan	Borovicka[3]
Remus	Frei[3]
Johannes	Haarer[3]
Sandra	Müller[3]
Mikael	Sawatzki[3]
Gian-Marco	Semadeni[3]

[1]Affiliation: Department of Visceral Surgery and Medicine, Inselspital Bern University Hospital, University of Bern, Bern, Switzerland

[2]Affiliation: Medical Clinic II, Ludwig Maximilian University of Munich, Munich, Germany

[3]Affiliation: Department of Gastroenterology and Hepatology, Cantonal Hospital St. Gallen, St. Gallen, Switzerland
